# Tribes and tribulations: interdisciplinary eHealth in providing services for people with a traumatic brain injury (TBI)

**DOI:** 10.1186/s12913-017-2721-2

**Published:** 2017-11-21

**Authors:** M. Hines, M. Brunner, S. Poon, M. Lam, V. Tran, D. Yu, L. Togher, T. Shaw, E. Power

**Affiliations:** 1 Faculty of Health Sciences, The University of Sydney, PO Box 170, Lidcombe, NSW 1825 Australia; 2 Faculty of Education and Arts, The University of Newcastle, University Drive, Callaghan, NSW 2308 Australia; 3 Faculty of Engineering and Information Technologies, The University of Sydney, Camperdown, NSW 2006 Australia; 4 Faculty of Health, University of Technology Sydney, Ultimo, NSW 2007 Australia; 5 Moving Ahead, NHMRC Centre of Research Excellence in Brain Recovery, School of Psychology, The University of New South Wales, Camperdown, NSW 2052 Australia

**Keywords:** Multidisciplinary, Electronic health record, Technology, Allied health, Team, Telehealth

## Abstract

**Background:**

eHealth has potential for supporting interdisciplinary care in contemporary traumatic brain injury (TBI) rehabilitation practice, yet little is known about whether this potential is being realised, or what needs to be done to further support its implementation. The purpose of this study was to explore health professionals’ experiences of, and attitudes towards eHealth technologies to support interdisciplinary practice within rehabilitation for people after TBI.

**Methods:**

A qualitative study using narrative analysis was conducted. One individual interview and three focus groups were conducted with health professionals (*n* = 17) working in TBI rehabilitation in public and private healthcare settings across regional and metropolitan New South Wales, Australia.

**Results:**

Narrative analysis revealed that participants held largely favourable views about eHealth and its potential to support interdisciplinary practice in TBI rehabilitation. However, participants encountered various issues related to (a) the design of, and access to electronic medical records, (b) technology, (c) eHealth implementation, and (d) information and communication technology processes that disconnected them from the work they needed to accomplish. In response, health professionals attempted to make the most of unsatisfactory eHealth systems and processes, but were still mostly unsuccessful in optimising the quality, efficiency, and client-centredness of their work.

**Conclusions:**

Attention to sources of disconnection experienced by health professionals, specifically design of, and access to electronic health records, eHealth resourcing, and policies and procedures related to eHealth and interdisciplinary practice are required if the potential of eHealth for supporting interdisciplinary practice is to be realised.

## Background

Increasingly, eHealth is an integral component of modern health care. eHealth embraces the application of information and communication technologies (ICT) to health care and encompasses a broad range of web-based and mobile health interventions, including the use of smart sensors and personal biofeedback devices as well as use of telehealth and electronic medical records (EMR) [1]. Enthusiasm for eHealth worldwide has been fuelled by its potential to revolutionise health care by maximising efficiencies and supporting optimal client outcomes through improved quality of care [2]. Consequently, governments and health organisations alike have invested significantly in eHealth strategies [3]. One promising area for eHealth research and development that warrants exploration is its application to interdisciplinary care.

Interdisciplinary teamwork is a particular hallmark of contemporary practice in traumatic brain injury (TBI) rehabilitation [4]. TBI occurs as a result of accident or trauma from an external force, and is associated with a broad range of associated impairments, including physical, neurological, cognitive, behavioural, and psychosocial sequelae [5]. The varied and complex nature of deficits secondary to TBI necessitates a diverse rehabilitation team incorporating health professionals from an array of disciplines, including medical, nursing, and allied health [6]. These interdisciplinary teams span the continuum of care, mirroring the transition of a person with TBI from initial admission to acute medical services through to hospital-, centre- and/or community-based rehabilitation programs [6].

Strong interpersonal communication across health providers and with consumers underscores effective interdisciplinary teamwork in health [7]. Over the past decade, the emergence of eHealth technologies have offered health professionals working with complex populations such as those with a TBI a new avenue to foster interdisciplinary communication. Allied health professionals have identified the potential benefits of eHealth to include improved collaboration and continuity of care across health care providers, as well as improved communication with consumers [3]. Use of the EMR in particular, has emerged as a strategy that may support improved coordination of care by facilitating efficient sharing of patient information between providers and across the continuum of care [3]. For instance, in Australia, the delivery of the My Health Record system by the Australian Digital Health Agency represents a key component of a national approach to improving health system efficiency [8]. Similarly, use of mobile and web-based platforms such as videoconferencing may help facilitate teamwork by bringing together disparate members of the interdisciplinary care team [3, 7]. To date, little is known about the use of eHealth for interdisciplinary practice for TBI rehabilitation, however emerging evidence suggests its potential for supporting quality care [9].

Despite the promise of eHealth, application of technology to health care contexts has not always resulted in improved interdisciplinary practice [7]. Successful implementation must build on a strong understanding of the readiness of health professionals and consumers to engage in eHealth technologies [3]. Currently, little is known about the nature of these barriers and enablers for contemporary TBI practice, and there has been minimal attention in the literature to organisational, policy, and clinical leadership factors that support implementation of eHealth across multiple organisations and across the continuum of care [9]. Nonetheless, eHealth is becoming a feature of TBI rehabilitation practice [10]. Recent systematic reviews have reported positive outcomes from the use of technology in rehabilitation after TBI [10–14], such as telehealth, smartphones, and social media. However, there is minimal research to date investigating treatment efficacy [11, 12], with the majority of studies being discipline-specific, rather than interdisciplinary eHealth practices. A clear understanding of the current state of eHealth implementation, and insight into the barriers and enablers for interdisciplinary practice is required to ensure provision of cost effective, efficient, and meaningful management of the complex challenges faced by people with TBI and their families. Consequently, the aim of this study was to explore experiences of using eHealth technologies to support interdisciplinary practice within rehabilitation for people with TBI from the perspectives of healthcare professionals (i.e., clinical, medical, and administrative staff) who utilise them. Specifically, we sought to understand healthcare professionals’ experience with, and attitudes towards, the uses of eHealth within the workplace, and the extent to which eHealth is successful in supporting interdisciplinary practice.

## Methods

### Research design

An interpretive qualitative study using narrative analysis was conducted involving data collected from focus groups and in-depth interview. Focus group and interview methods [15, 16] were chosen to explore shared and divergent perceptions and experiences of using eHealth in interdisciplinary clinical practice [17]. The design and reporting of the study were informed by the Consolidated Criteria for Reporting Qualitative Research (COREQ) guidelines involving interviews and focus groups [18].

### Participant recruitment

Ethical approval for the research was received from the Human Research Ethics Committee at the University of Sydney (Project number: 2014/1017) and the organisations that assisted with recruitment (details removed to protect participants’ and organisations’ anonymity). The primary focus of this research was to examine the current role of eHealth in supporting interdisciplinary practice within rehabilitation for people with chronic health conditions. A single domain, i.e., rehabilitation after TBI, was chosen as an exemplar for this exploratory research due to the interdisciplinary nature of TBI rehabilitation and the potential benefits that eHealth may have in this domain [19].

Four healthcare organisations in New South Wales (Australia) known to provide TBI rehabilitation services were identified based on purposeful sampling techniques [20] in order to ensure that we captured a range of diverse perspectives on eHealth use in TBI rehabilitation. That is, organisations contrasted in terms of their geographical location (metropolitan or regional) and funding sources (publicly or privately funded services). A known staff member within each organisation’s TBI rehabilitation teams was invited to distribute information about the study with other members of the TBI teams who met the study’s inclusion criteria: (a) an adult over 18 years of age, (b) a member of a TBI rehabilitation team in a healthcare or administrative role, and (c) currently using eHealth technologies to provide health services to individuals with TBI. Information sheets included a brief description of the purpose of the study, including the voluntary nature of participation. Individuals who were interested in participating indicated their interest to the key contact person in their organisation and completed a written consent form.

### Data collection

Four key questions were used to explore a range of experiences of, and issues related to eHealth for interdisciplinary practice, and to facilitate discussion between participants in focus groups (see Table 1). Questions were open-ended and aimed to elicit discussion that uncovered participants’ experiences, challenges faced, and attitudes towards the use of eHealth in interdisciplinary healthcare. These were derived from a systematic review recently conducted by the research team [9], and surveys that have been utilised in eHealth research [3]. As required, additional sub-questions, prompt questions, and visual prompts (e.g., key phases in the continuum of care, healthcare processes that can be enabled by eHealth) were provided to ensure discussion was relevant to the research questions.

**Table 1:** Focus group / Interview Questions

1.	We would like to get a snapshot of how you work as a team along the continuum of care. a) Who are the members of your interdisciplinary team? b) What interdisciplinary processes are in place along the continuum of care? c) What has worked well and why? d) What hasn’t worked so well and why?
2.	We now want to look at how eHealth may support interdisciplinary practice along the continuum of care. a) What are your experiences of using eHealth? b) What devices/technology have you used? c) In using eHealth to support interdisciplinary practice, what has worked well? Why? d) In using eHealth to support interdisciplinary practice, what hasn’t worked so well? Why? e) What has been the clients’ response to using eHealth?
3.	We have discussed how eHealth is being used to support interdisciplinary practice now. How would you like to see eHealth enhance interdisciplinary practice for clients? a) What areas along the continuum of care need support? b) If you could have anything - what would it look like? (BLUE SKY)
4.	Wrapping up: a) Of all the things we have discussed, which is the most important to you? b) Give short oral summary of focus group – Is this an adequate summary? c) Is there anything else that we missed that you think we should know?
Prompts	• Point of focus is interdisciplinary teamwork and how eHealth can support this in practice • Is this specific to your profession/discipline or does it relate to interdisciplinary team?

Each focus group or interview was allocated one and a half hours of time and conducted at the participants’ work places, which included offices in hospitals in metropolitan and rural settings, facilitated by a primary moderator, an assistant moderator, and one or two research assistants. All researchers present took notes on the discussion and seating arrangements of participants. Discussion was digitally recorded and later transcribed verbatim, with transcripts de-identified and checked for accuracy against the digital recording by the researchers. The final transcript and a summary of key discussion points were emailed to participants for verification of content and concepts.

### Data analysis

Narrative analysis, with its attention to sequence and consequence [21] was selected as a fitting approach as it allows participants’ accounts to be understood as a whole, rather than fragmented and disconnected into smaller thematic categories. Consideration of narrative elements used in participants’ accounts according to Labov’s [22] framework provided insights into participants’ perspectives, attitudes, and experiences with eHealth. This structural analysis was coupled with a thematic analysis of the narrative contents discussed by participants [23]. Patterns and meanings conveyed by participants were then compared and contrasted within and across interviews and focus groups to identify common and divergent themes, experiences, and perspectives, and facilitated identification of counter-narratives which contrasted with the majority of narratives on a particular topic. This facilitated insights into the impact of differences in geographical location, organisation types, and participants’ individual disciplines and roles within their respective TBI rehabilitation teams on their experiences with eHealth. Since interviews and focus groups were semi-structured, not all participants were asked all questions in exactly the same way, and every participant within each focus group may not have had the same opportunity to provide a response to specific questions. In these situations, quantification of statements may misrepresent the data [24]. Thus, in this paper, we have not provided precise quantification of the numbers of participants who described a particular topic or theme, but rather have used terms such as “a few”, “some”, and “most” in order to highlight the degree to which statements and patterns were typical, or were unusual across the participant group.

Six authors (MH, MB, ML, SP, DY, VT) met to discuss initial impressions of the data and identify key topics and themes. Subsequent data analysis was primarily conducted by three authors (MH, MB, EP). Following preliminary analysis, the first author independently applied initial codes to the raw data. A first pass at grouping the data according to an overarching narrative structure was discussed by the first and final authors to ensure consensus and reliability in the development of themes. The grouping and discussion process continued using NVivo® with the first author revising and regrouping the data until a logical sense of the data and a comprehension of the final themes could be reached. The final themes were then reviewed to ensure that consensus was met regarding how core meanings expressed by participants were interpreted.

## Results

### Participants

Seventeen participants took part in the study, with three focus groups and one individual interview conducted between August 2015 and June 2016. The participants were predominantly allied health professionals working in rehabilitation teams in regional and metropolitan areas of NSW. The three focus groups consisted of participants that worked in three independent, interdisciplinary teams. Focus Groups 1 and 2 (FG1, FG2) were based in regional NSW, and Focus Group 3 (FG3) was based in metropolitan Sydney. All participants provided public rehabilitation services, and two provided privately funded services for people with TBI. The individual interview was conducted with a private practitioner who provided rehabilitation services in metropolitan Sydney. A summary of participant demographics is provided in Table 2. No requests for alteration of transcripts or summaries of key discussion points were received from participants. In this paper, participant numbers are used to refer to individual participants; individual participants’ occupations and role within the TBI rehabilitation team are not provided where this does not provide additional insights into their perceptions, in order to protect confidentiality.

**Table 2:** Summary of participant demographics

	FG1^a^ (*n* = 5)	FG2 (*n* = 6)	FG3 (*n* = 5)	Interview (*n* = 1)
Region	Regional	Regional	Metropolitan	Metropolitan
Organisation type	Non-government organisation	Government organisation	Non-government organisation	Private Practice
Services provided	Public & Private inpatient, outpatient, and community rehabilitation	Public community rehabilitation	Public & Private inpatient, outpatient, and community rehabilitation	Private inpatient and community rehabilitation
Participants’ occupation				
Allied health	4	5	5	1
Medical	1	–	–	–
Administration	–	1	–	–
Participants’ age				
>50 years	1	3	–	1
41–50 years	1	1	–	–
31–40 years	1	2	3	–
21–30 years	1	–	2	–
Unspecified	1	–	–	–
Participants’ years working in current team				
>10 years	1	3	–	1
5–10 years	–	–	1	–
1–5 years	3	2	3	–
<1 year	–	1	1	–
Unspecified	1	–	–	–

^a^FG = Focus Group

### ‘Disconnection’: The common narrative plot

There was an overarching theme in the common narrative plot, one of disconnection. For example, regardless of their specific geographical and organisational setting, participants readily recognised the potential of eHealth for TBI interdisciplinary rehabilitation. However, they described confronting many barriers in their use of eHealth which disconnected them from achieving the quality of care that they wanted to in their work, affecting their ability to realise this potential. Disconnections were particularly evident in activities and interactions between different ‘tribes’ involved in health care, i.e., between disciplines, departments, or organisational settings. In the majority of narratives, participants took various actions to make the most of available eHealth technologies, but these still usually resulted in compromised quality, efficiency, and/or client-centredness. In only a minority of cases, participants described narratives that directly contrasted with this common narrative plot. In these instances, the unifying theme was one of connection, as participants described successfully achieving the potential of eHealth.

**Fig. 1:**
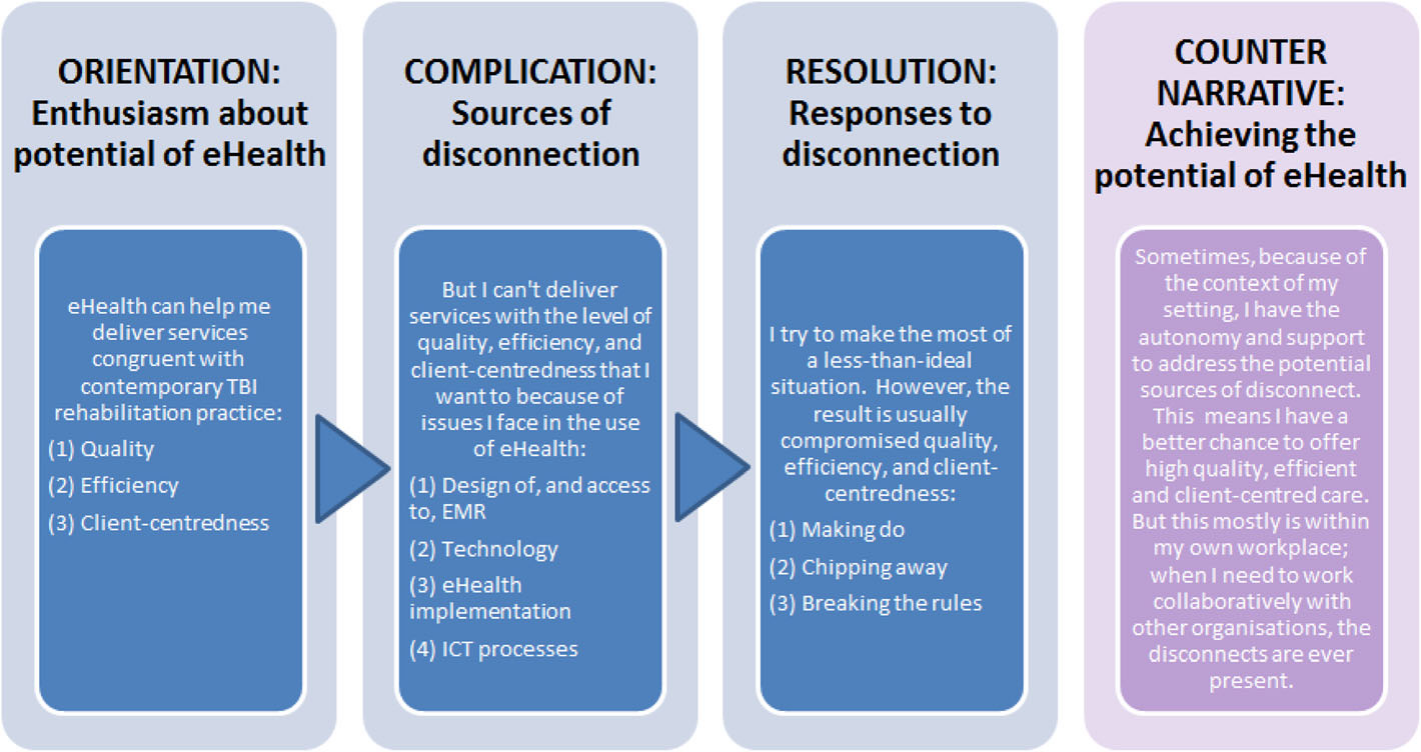
The common narrative plot and its counter-narrative

Figure 1 presents an overview of the common narrative plot in the collective participant voice, namely: (a) orientation: enthusiasm about the potential of eHealth; (b) complication: sources of disconnection; (c) resolution: responses to disconnection; and (d) the counter-narrative: achieving the potential of eHealth. Below, we provide a detailed exploration of each narrative component in sequence.

*We do a good job, but we’ve got a potential to do a magnificent job… It’s not that we’re not willing to … go with the times, we can’t right now, we don’t have the systems... So it’s not the therapists being the barrier to progression.* (P4).

### Orientation: Enthusiasm about the potential of eHealth

All participants described eHealth as having the potential to align closely with the aims of contemporary TBI practice, namely, to provide (a) efficient, (b) client-centred, and (c) quality services. Participants said that their work required them to collaborate with team members across the continuum of care, and professionals from other organisations (e.g., publicly-funded acute hospital settings, rehabilitation centres, non-government organisations, private practices, and funding bodies). A key benefit of eHealth was described as its potential to support efficient and timely communication and transmission of client information, including with team members who are otherwise difficult to communicate with in-person, such as those who work part-time or in external organisations, or who provide services outside the clinic environment. The immediate access to client information afforded by EMR systems was valued as it eliminated the need to spend substantial amounts of time chasing up information, improved case coordination, and supported timely clinical decision making. One participant (P4) favourably compared the timeliness of referral information received via eHealth as compared with mailed written reports, based on its impact on patient outcomes. She commented that without access to an EMR, *“You might receive [a swallowing referral report] four weeks later, and [the patient is] are currently in [hospital] with their pneumonia.”*

Participants in each focus group and interview expressed the belief that eHealth aligned with client-centred practice. Participants said that eHealth was consistent with how people communicate and helped clinicians approach *“information in the same way that the rest of the world does these days.”* (P12) Accordingly, participants believed that eHealth supported functional approaches to therapy recommended for contemporary TBI practice. Some participants also reported that eHealth could help to address some of the critical deficits associated with TBI; for instance, clients with impaired memory benefit from eHealth strategies such as automated SMS appointment reminders, whilst those with attention deficits may benefit from frequent sessions of shorter duration facilitated via videoconferencing.

Although all participants were largely supportive of integrating eHealth in interdisciplinary practice, some identified the need for a critical approach to account for perceived potential limitations associated with the medium. For instance, some participants in FG2 and FG3 were uncertain that the quality of clinical notes recorded electronically compared favourably to pen-and-paper methods. Most participants did not describe themselves as proficient with technology. Some mentioned that a lack of eHealth training in university programs, coupled with the ongoing evolution of health technology, meant that they had a limited understanding of potential applications of eHealth to their work, and they continued to require support in its use.

### Complication: Sources of disconnection

Participants’ narratives typically included a sequence of events and complicating actions that hampered their ability to successfully incorporate eHealth into their work, and left them feeling disconnected from achieving quality of care in their work. Four broad sources of complicating actions, or barriers to effective eHealth use were recounted: (a) design of, and access to, the EMR; (b) technology; (c) eHealth implementation; and (d) ICT processes. Each source is described in detail below.

#### Design of, and access to, EMR systems

Complications relating to the use of EMR dominated eHealth narratives. The design of EMR systems was invariably described as inadequate as they failed to provide timely access to required clinical information. For example, although contemporary community based TBI practice was described as involving decentralised teams, EMR systems were only accessible to team members within the same organisation. Being organisation-centred rather than client-centred in design, firewalls in EMR systems precluded access to team members from outside the organisation, the health district, or public health agency, even when clients were referred from these organisations. This was particularly a prominent feature of complications described by participants from outside the public health sector. Participants believed this was inconsistent with principles of interdisciplinary practice: *“What’s the point? It's either it’s a team or it’s not a team.”* (P1) Another participant from a non-government organisation (P2) described lack of access to the public health system EMR, despite receiving training in its use:

*But we cannot access that system here. Our information system doesn’t talk to the NSW Health system. There’s a big firewall. So the systems they would like us to use, they don’t let us in to use them.*

Aside from creating unnecessary inefficiencies in chasing up clinical information, participants, particularly those working outside the public health sector, stated that lack of EMR access potentially compromised the quality of care. In the absence of reliable eHealth strategies such as an accessible EMR, reliance on direct communication (e.g., in person, telephone) with previous therapists, for instance, was described as inadequate in ensuring that necessary information was shared across the entire team.

*Obviously that falls down if people aren’t available or busy and not responding to emails, on leave, all that stuff... It does mean it has potential for things to get lost in the ether.* (P16).

Lack of integration between multiple EMR systems was also major source of inefficiency, as *“nothing talks to each other.”* (P8).

*If I want to find out what’s happened to a client within our own local health district, I’ll look at three places… And each medical record has separate access with a separate log in. And then if you want to find out what’s happening in [an acute hospital], that’s when you do the medical records check. I can do it quickly now, I’ve mastered the art, but new team members might look at EMR but they don’t think to look at [another EMR system].* (P9).

Lack of access to any EMR systems was reported by some participants in FG2 who said this impacted on the wider team: *“We only do paper files in this service. So we don’t do any electronic medical record officially… and people find that incredibly irritating in the rest of the health service.”* (P9).

Those participants who did have access expressed dissatisfaction with the usability of EMR systems due to their limited functionality. Participants reported that they were unable to upload client data in the form of electronic documents containing test results, photographs of wounds or house modifications, and videos of physical tests conducted during sessions. Instead, client records were text-only entries with limited formatting options such as bulleted lists and headings, resulting in unnecessary duplication and inefficiency.

##### Counter-narrative: Achieving the potential of eHealth with EMR

The narratives of experiences with EMR systems from the private practitioner (P1) and one focus group contrasted noticeably with others. In both settings, clinicians had direct involvement in the design of the EMR systems. For instance, the private practitioner was able to draw on the ICT expertise of a colleague to design a customised EMR and administration system for use within her own practice. Although still lacking access to other organisations’ EMR, this fit-for-purpose system maximised efficiencies within her own practice, and overcame many sources of disconnect described in other focus groups. Similarly, participants in this focus group were part of the development of a new organisation-wide EMR system, and actively involved in trialling functions in a test environment and identifying issues needing to be addressed. As a result of this ongoing improvement process, participants said that the EMR increasingly reflected their needs. These counter-narratives of positive EMR experiences were consistently missing from the other focus groups.

#### Technology

Insufficient access to required technology, including the lack of access to mobile devices such as tablets and laptops for use in clients’ homes or in the community, created additional complications in eHealth narratives across all focus groups, but not in the interview with the private practitioner (P1). Even when mobile devices were available through their employer organisation, these were often older models and inadequate for intended clinical tasks. Participants in FG2 related:

*Given that we’re mostly away from this building … and we do not have iPads or laptops or other things to make us connected when we’re out in the community, that’s got some issues… We’ve got mobile phones but they don’t work everywhere in the region.* (P7).

*And they’re not iPhones.* (P10).

*The old brick, yeah… They make calls and send texts and that’s it.* (P7).

Similarly, access to videoconferencing facilities was variable and at times insufficient to meet a range of clinical tasks. Whilst participants in FG3 were satisfied with their access to quality videoconferencing facilities, participants from FG1 reported that their organisation did not have such facilities, and alternatives were inadequate: *“The closest one is the videoconference room at the [hospital], but I wouldn’t call it a patient-accessible room because you go through a staff kitchenette. It’s for staff. It’s not set up for a direct client service.”* (P4).

Insufficient access to the internet and mobile networks was also described as a complicating factor in eHealth narratives of participants from regional areas. Variable coverage across their districts affected opportunities to utilise eHealth strategies in the clinic and the community. When describing the inconsistency of internet connectivity at work, one participant (P4) said: *“Our Internet doesn’t like Fridays.”* She later described the difficulty of establishing reliable videoconferencing services with clients from small rural towns: *“You need the major infrastructure for speed and for reliability of service – and it’s not there.”* Moreover, the prohibitive cost of internet usage in regional and rural areas meant that telehealth services were simply deemed unfeasible for many clients.

Participants from metropolitan and regional areas alike reported insufficient system and server capacity. Some reported intermittent crashing of EMR systems, whereas others were discouraged from storing audio and video files on the server due to the limited capacity for storage. *“We’re supposed to be going all electronic but they’re not giving us the capacity to be able to save the electronic stuff because they keep telling us that the server’s too small.”* (P8).

#### eHealth policy implementation

Complications related to eHealth implementation also emerged as a major source of complicating actions and disconnection for participants.

##### Restrictive and inconsistent eHealth policies

Across all focus groups and the individual interview, restrictive eHealth policies prevented participants from engaging in numerous eHealth activities. Participants in FG1 said they were not allowed to send and receive text messages from work mobile phones, and needed to strenuously justify requests to purchase the latest smartphone models, rather than superseded technology. Although participants identified potential uses for social media and video sharing sites in their work, participants in FG1 and FG2 reported that access to these platforms was generally restricted. One participant (P10) remarked: *“There’s a whole heap of technology that could be used but we can’t. So for example, we’ve got blocks on YouTube which you use across the board, from therapy to information, education and also learning for staff.”* Participants identified that such restrictive policies were at odds with person-centred practice: *“People forget that people walk outside the health service doors sometimes.”* (P9).

Similarly, policy blocks on cloud-based computing thwarted participants’ attempts to share clinical information with team members in external organisations as a way of circumventing restricted access to EMR systems. Participants in FG1 reported blocked access to Wi-Fi and no ability to authorise additions or updates on tablets, resulting in a complicated process to download therapy applications and use photographs in client resources: *“If we want to get more apps on it, say we have a new client, we have to send it back to [the ICT department 6 hours away]. We don’t have access to the password for iTunes.”* (P5) Another participant (P4) later reported: *“Another time, we wanted to use it to take photos to create low-tech communication aids. But we couldn’t email photos from the iPad to print them.”*

Experiences with restrictive eHealth policies extended to electronic communication. Some participants in all focus groups and the individual interview noted that due to privacy concerns, policies permitted electronic sharing of clinical information only within the organisation, which created complications when information needed to be shared with external organisations and funding bodies. Participants also identified inconsistencies in how policies were applied. For instance, although electronic sharing of information with external team members was restricted, it was noted that clinical handover and referral information was received or accepted from these external organisations via email. Electronic communication policies were reported to vary widely across funding bodies, with some specifically requiring communication in electronic format while others would not utilise email for the same purposes.

A feature of restrictive eHealth policies that emerged during focus groups was their arbitrary implementation. In the course of the discussion,participants in FG1 and FG2 were surprised to discover that other team members were able to access platforms that they were barred from, such as desk-based messenger services, text messaging, and systems for secure transmission of client information. These inconsistencies were interpreted by some participants as being based on differential power and status of team members. For instance, when it emerged that one participant, a doctor, could access Wi-Fi, an allied health professional (P4) remarked: *“Plebs can’t access Wi-Fi.”* In fact, restrictive or inconsistent policies were seen as indicating that management did not understand how eHealth could be applied to clinical tasks, and did not trust clinicians to be professional and trustworthy in its use. One participant (P8) remarked: *“It’s like instead of treating us like we’re all professional people, you’re treated like children who can’t do that. If you have internet access, you’ll be on the porn sites.”*

##### Lack of eHealth policies and procedures

In some cases, participants identified lack of clear policy or procedures for eHealth including minor issues such as procedures for purchasing and downloading therapy applications, through to critical issues, such as procedures to ensure safe transmission of client information. Participants in all focus groups and the individual interview were either unaware of specific organisational procedures, or were further confused by the variable approaches to electronic communication enforced by funding bodies and service providers. One participant (P14) commented: *“I’ve not read a formal policy around [electronic transmission of client information] but it doesn’t necessarily mean there’s not one.”*

##### Lack of inter-organisational approach to implementation

eHealth policies and procedures were typically described by participants from each focus group and the individual interview as organisation-centric, rather than designed around individual clients and the wider TBI rehabilitation team and the resources available to them. For example, participants said that organisations utilised various videoconferencing platforms, some of which were not accessible to team members in external organisations. Similarly, participants in two focus groups said that videoconferencing policies in their organisations required a staff member to support clients on the remote end, which was problematic as these resources were often not available in external organisations. One participant described a failed attempt to arrange videoconferencing for a physiotherapy consultation, culminating in the client changing their service provider. This disconnection also extended to access to web-based professional development and training materials. Although participants from within the public health sector described uncomplicated access to online training modules, those from the non-government organisation reported that some modules required a public health staff log-in, resulting in lack of access to training they were expected to complete.

Communication of eHealth policy changes also reflected the same narrow, organisation-centric focus. Although policies about electronic communication of client information had implications for team members in external organisations, narratives demonstrated that communication of policy changes did not take them into account. In describing a change in hospital policy on accessing test results, one participant (P2) from outside the public health sector reported: *“Before they [an external organisation in public health sector] used to fax pathology and particularly imaging reports from X-rays, they’d automatically fax them. And they stopped doing that. And we didn’t know that they were expecting us to check it online.”*

Failure to consider the wider team in eHealth implementation resulted in efficiencies not being realised equally across the team. For example, whilst sending photographs of a client’s wound via teleconference enabled collaboration with metropolitan-based specialists, efficiency was significantly compromised for the rural clinician (P6) who was required to travel to the client to take the photo, in line with standard procedures. She described: *“They always want photos of the wound, so that recently that involved travelling out to [a town] to the patient, just a couple of hours’ drive, taking a couple of photos.”*

##### Counter-narrative: Achieving the potential of eHealth through flexible policy implementation

Only a minority of participants described policy implementation as being supportive of their implementation of eHealth. Specifically, participants from FG3 were satisfied with their organisation’s more liberal stance on emailing clients and family members as an efficient means of communication, and did not encounter the barrier of restrictive policies on electronic communication as described by other participants. Although participants from FG3 said that clients were occasionally inappropriate in their use of email with staff, they believed this was to be expected, and straightforward to address: “*People cross boundaries and you just give them feedback and that’s fine… which is part and parcel for anyone working in brain injury.”* (P13).

#### ICT processes

A final source of complicating action and disconnection emerged from the onerous nature of processes to address ICT and eHealth problems. Participants in all three focus groups reported that decisions regarding purchase of equipment and access to eHealth systems required involvement and approval of multiple individuals, yet these difficulties were noticeably absent in the narratives of the private practitioner. One participant (P4) stated: *“We might ask the question, but then they [the ICT department] will wait until you have a health service manager engagement in to go, yes, can you please do that job for that clinician. So it’s just fiddly.”* In other cases, due to restrictive eHealth policies and procedures, seemingly straight-forward tasks required elaborate actions to resolve them. One participant in a regional area reported that in order to download therapy applications on a tablet for a new client, she needed to send the tablet to a metropolitan city where the ICT team was based, as she did not have the required permissions.

Convoluted decision-making pathways were further complicated by communication difficulties experienced between TBI rehabilitation and ICT staff, a major barrier being that ICT staff were often not perceived to understand clinicians’ roles or priorities, and the implications of eHealth for their work. For example, one participant (P14) remarked: *“I don’t know they necessarily always understand all the priorities sometimes that we place on some aspects of things so it can take a little while to get aspects of things actioned.”* Participants conceded that communication problems were two-sided, as they had difficulty clearly and accurately expressing their needs: *“Part of the problem is that I don’t speak IT language.”* (P3) Ultimately, onerous processes and ineffectual communication resulted in a lack of timeliness in resolving eHealth problems.

##### Counter-narrative: Successful communication with ICT staff.

Although most participants related difficulties communicating in a clear and efficient way with ICT staff, a minority described communicating about eHealth problems with colleagues who had expertise in both ICT and clinical tasks. Consequently, these participants were much more satisfied with ICT processes, and the timeliness with which issues were addressed.

### Resolution: Responses to disconnection

In response to the array of factors that disconnected participants from using eHealth for interdisciplinary practice, participants recounted resolutions to their narratives that involved a range of actions to make the most of less-than-ideal circumstances: (a) making do, and in extreme cases, giving up; (b) chipping away; and (c) breaking rules. However, in most cases these responses were largely inadequate in supporting quality and efficient client-centred care.

#### Making do

In the majority of cases for participants in all focus groups and the individual interview, the resolution of eHealth narratives involved participants persisting and putting up with existing systems and processes. Although aware that this resulted in compromised efficiency and quality of care, participants focused on their ability to achieve basic work roles. One participant (P12) summed up the situation:

*I guess you make do with what you’ve got, and so is it enough for us to do our job now? Yes, because we’re getting on and doing our job. Are there things that would make things a lot more efficient?... Obviously there’s a time and effort saving in there. I guess we muddle along the best we can with what we have.*

At times, persisting with inefficient eHealth systems or processes required problem solving and development of other means to complete necessary tasks. For instance, participants who were unable to access the videoconferencing platforms used by the interdisciplinary team utilised the telephone instead. Similarly, when unable to access the required clinical information via EMR systems, participants sought to gain such information from other team members via email, telephone, and fax. Participants working outside the public health sector even reported physically travelling to external organisations to borrow clients’ physical files, access computers to view client notes, or to speak in-person to team members in external organisations. Yet, difficulties making in-person contact with busy team members further exacerbated inefficiencies. Participants regularly stated that they needed to develop strong professional relationships with health professionals across a range of organisations in order to ensure they received the clinical information they required. For instance, when describing communication with medical doctors, one participant (P17) said: *“You become best mates with their receptionist because you can never talk to the specialist to find out what's going on.”* She later remarked on the inefficiencies involved in communicating with private practitioners:

*I would love it if there was a program created that every private therapist that we had to work with we could say, “You join this program,” and they could see all of the notes and our reports and everything that had to happen so we didn’t have to make a thousand different phone calls to different providers saying, “I need this, I need this, I need this, I need this,” that would could all just see the one thing… Circumvent all those multiple phone calls and chasing information and all that stuff.*

In a minority of narratives, participants described ‘giving up’, as they were unable to identify alternative solutions and deemed sources of disconnect as insurmountable. For instance, one participant (P2) said he returned equipment to his organisation as he believed he would be unable to satisfactorily resolve eHealth issues:

*A lot of my junior colleagues use a lot of the iPhones to do Skype and take pictures and send them to the clients. So when I was getting a replacement phone here I’d asked for a smart phone, so I was given an iPhone. But there are so much restrictions, I could not download any apps, not one to use Skype. So I actually gave it back.*

#### Chipping away

At other times, participants attempted to make incremental improvements to eHealth systems and processes by advocating for change to management and/or ICT departments. However, change was generally not achieved in a timely manner, mainly as a result of ineffectual communication with management and ICT and onerous decision making processes. For instance, one participant said it took years of negotiation for him to secure access to an external organisation’s EMR system. As a result, ‘chipping away’ at inefficient or ineffective eHealth systems and processes required persistence and tenacity on the part of participants. One participant (P5) remarked: “*We just chip away at it. We just keep asking and asking.”*

#### Breaking rules

On occasion, participants judged the disconnection caused by existing eHealth systems and processes as threatening the quality and efficiency of their work to an unreasonable degree. Under such circumstances, some participants, particularly those working in government and non-government organisations where policy restrictions were more onerous, described ‘breaking rules’ – organisational policies and procedures – in order to achieve the work they needed to do in an effective, efficient manner.

One source of rule-breaking involved the use of participants’ own devices, data, and funds to circumvent sources of disconnection such as lack of access to technology, onerous ICT processes, and restrictive policies and procedures, in order to achieve quality, efficient, and client-centred services.

*If there was an app that I really found was going to help my work, if there was a way for me just to load it on here and use my own money to pay for it, and I’d just claim it in tax, I’d be fine to do that.* (P17).

Participants acknowledged that this course of action failed to achieve sustained change. One (P12) remarked: *“I’d just take my iPad and go and do what I got to do which is head in the sand, it doesn't actually progress the cause in anyway.”*

Rule-breaking was particularly apparent for addressing conflicting policies about electronic communication of client information with external organisations. When describing the ease of electronic communication, one participant (P6) remarked: *“It works great, but we don’t talk about it too much because the policy here is you don’t email much.”*

Before deciding to disregard organisational eHealth policies or procedures, participants described assessing the risk involved as minimal, and were often unable to identify alternative courses of action.

*I’ve taken the view that [this funding body] is another government agency, it will have its own good firewalls, it’s probably pretty safe, but theoretically every time I do that, I am breaching the guidelines. And yet if we didn’t do it that way, how would we do it?* (P9).

Other comments suggested that participants were not always completely aware of the risks entailed in contravening policies. For instance, although participants saw little harm in using their own devices, others raised concerns about security and privacy risks. One participant (P17) described her feelings about access to mobile devices at work:

*I think clinicians are really happy that they can take photos and videos without using their own phone, and then having to be paranoid, have I wiped that off my phone? Because I certainly hated taking photos with my phone because it goes onto my husband’s phone as well so I would be so paranoid that, okay, I need to email that to myself and wipe it.*

## Discussion

In this study, we sought to gain insights into health professionals’ experiences with eHealth technologies to support interdisciplinary practice in TBI rehabilitation. Consideration of participants’ narratives revealed that, on the whole, the potential for eHealth to support interdisciplinary practice has not been realised. Instead, health professionals revealed that they felt disconnected from achieving quality of care to the degree that they wanted to, particularly when this work involves working with team members from different ‘tribes’, such as in other disciplines, departments, or organisations.

Whilst health professionals in our study readily identified the need to develop additional skills and knowledge related to eHealth, they were notably enthusiastic about its potential. These findings suggest that positive health professional attitudes may be promising facilitators of future eHealth adoption. Yet, as the current findings suggest, positive attitudes alone are not sufficient to drive successful implementation. Health professionals may resist use of eHealth systems that they perceive to conflict with their professional values and work roles, even if they are otherwise favourable to technology [25]. Provision of training in eHealth will therefore not necessarily be effective in driving uptake because of a range of other barriers beyond the domain of individual health professionals. In fact, there is a risk of threatening or destabilising these positive attitudes if these sources of disconnect are not satisfactorily addressed, as simplistic solutions within complex systems can produce unexpected outcomes [26].

Consideration of health professionals’ responses to disconnections in using eHealth for interdisciplinary practice underscores the necessity to resolve these issues and avoid risks associated with unintended consequences. In the current study, health professionals described various adverse consequences on the quality, efficiency, and client-centredness of the rehabilitation services they deliver. Addressing sources of disconnect may also help to circumvent the ethical implications of clinicians’ well intentioned responses to these barriers, such as taking action without a full understanding of the degree of risk entailed to themselves, their employer organisations, and their clients, or by personally funding their own eHealth use at work.

The most striking source of disconnect encountered in this study related to the design of, and access to the EMR, specifically, the inability to facilitate clinical tasks by making relevant patient date available. Our results suggest that in order to facilitate interdisciplinary practice involving all health professionals involved in TBI rehabilitation, access to the EMR is required across the continuum of care, and across public and private settings. Designing EMR systems that are centred on clients, rather than on single organisations, may facilitate integrated care [27], and support the fluid access to information desired by health professionals in this study. Internationally, there is increasing recognition of the value of providing patients with access to their own electronic health records [28]. For example, the Australian My Health Record [29] provides consumer access to their own digital health records, as well as to any health providers that they consent, via a single electronic system. Such systems may address some of the major concerns expressed in this study, such as issues related to firewalls and lack of integration between systems. By ensuring that all team members have sufficient access to the EMR, including viewing and editing rights, health professionals working in TBI rehabilitation may be supported to achieve integrated client care.

Evidence from the current study supports the claim that involvement of users in EMR design improves usability [30]. In this study, consideration of counter-narratives suggested that EMRs can best meet health professionals’ needs if they reflect an understanding of their clinical roles and priorities, which may be enabled by active involvement of health professionals in planning, design, and implementation. Health professionals, in particular, want increased functionality of the EMR, including the ability to upload a range of file types to the client record, in order to maximise the quality and efficiency of their work. This underscores the importance of enabling close alignment of the technical, social, and organisational dimensions of eHealth implementation, ensuring systems are useable, useful, and introduced appropriately by health organisations [25]. Consequently, implementation should not follow prescriptive approaches, but rather needs to be managed carefully, tailored to the unique set of dimensions for each healthcare setting [25].

Aside from addressing EMR design and access issues, our results demonstrate that additional attention to the wider resourcing of eHealth and the underpinning policies and procedures is needed. At the simplest level, organisations may need to ensure that their employees have access to updated mobile devices and videoconferencing facilities. Of fundamental importance is access to Wi-Fi and file sharing platforms that facilitate interdisciplinary practice with team members outside the organisation’s walls. Yet, based on the evidence from this study, it is likely that such resourcing and infrastructure will be insufficient unless also coupled with procedures and policies that correct prevailing restrictive approaches to eHealth, and that promote a person-centred, inter-organisational focus. Ensuring that supportive procedures and policies are in place may enable health professionals to achieve quality service provision, and may help to prevent the unintended consequences of rule-breaking and ‘making do’ with inadequate and inefficient eHealth systems and processes.

### Limitations of the study

In this study, we did not aim to achieve data saturation. It has been suggested that at least three or four focus groups per stratum is required before saturation can be ascertained [16]. Given the exploratory nature of this research, and our intention to sample participants from across a range of locations and practice settings, achieving data saturation was beyond the scope of this initial work. The participants in this qualitative study were all health professionals working in TBI rehabilitation in New South Wales, Australia, predominantly in allied health roles. It is therefore not known the extent to which findings in the current study apply to the professionals working with people with other chronic health conditions, or in other geographical locations. It is likely that future research incorporating additional participants working both within TBI rehabilitation as well as across a wider range of contexts may provide additional insights into eHealth-enabled interdisciplinary practice.

## Conclusions

The results from this study provide important insights into the extent to which eHealth is delivering on its promise to support interdisciplinary practice. Health professionals are largely supportive and enthusiastic about eHealth, but this is unlikely to translate into increased adoption of ICT and ultimately, positive client outcomes, unless urgent attention is paid to sources of disconnect in its use as reported by health professionals. Conversely, by rectifying issues related to the design of and access to the EMR, and with wider resourcing of eHealth and the development of supportive policies and procedures that underpin eHealth and interdisciplinary practice, service organisations may be better placed to support health professionals to deliver optimal client care.

## Abbreviations

EMR: Electronic medical records
FG: Focus group
ICT: Information and communication technologies
TBI: Traumatic brain injury
